# Reviewed article: Bezerra AB, Barrozo LV, Queiroz AP. Congenital heart diseases: population-based cross-sectional study and spatial analysis, São Paulo, 2012-2022. Epidemiol Serv Saude. 2026:35;e20250519

**DOI:** 10.1590/S2237-96222026v35e20250519.a

**Published:** 2026-03-16

**Authors:** Selma Alves Valente do Amaral Lopes

**Affiliations:** 1 Universidade Federal da Bahia, Salvador, BA, Brazil Universidade Federal da Bahia Salvador BA Brazil

## Peer review A

## Questionnaire

Does the manuscript contain new and significant information to justify publication? Yes

Does the Abstract (Summary) clearly and accurately describe the content of the article? Yes

Is the problem significant and concisely stated? Yes

Are the methods described comprehensively? Yes

Are the interpretations and conclusions justified by the results? No

Is adequate reference made to other work in the field? Yes

## Manuscript Structure

Length of article is: Adequate

Number of tables is: Adequate

Number of figures is: Adequate

Please state any conflict(s) of interest that you have in relation to the review of this paper. None

Interest: Good

Quality: Average

Originality: Good

Overall: Average

Recommendation: Major Revision

## Comments

A consideration for the authors is as follows:

The study is intriguing. It is technologically sophisticated and meticulously structured, and the subject matter is pertinent. However, two conceptual issues or pillars have not been considered or are mistaken in the collection and analysis, and these could compromise the authors’ analysis and interpretation of their results. 

1. The exclusion of newborns for whom no record of congenital anomalies of the cardiovascular system (ACSCV) was made would be an appropriate measure for this type of study. It is important to note that not all pregnant women undergo echocardiography (ECHO), and the absence of information detailing whether prenatal ECHO was performed or not on DNV records precludes the possibility of cross-referencing, for instance, data from Sisprenatal or SIM with data from SIH linked to SINASC or SIM. The newborns the authors considered as controls did not necessarily undergo screening for congenital heart disease. Importantly, the rate of detection of ACSCV from fetal ECHO is highly variable, and postnatal ECHO is required for definitive diagnosis, especially for minor ACSCV. In summary, the allocation bias is significant for the “control” category and should not be dismissed or justified by factors such as “underreporting of mild cases, lack of diagnosis at birth, and collection limitations.”

2. In the methodology, the authors specify that the records were “data from live births resident in the

municipality of São Paulo between 2012 and 2022.” However, when verifying the data from SINASC SP, it is evident that the data the researchers analyzed was entirely related to “occurrence” versus “residence.” This difference results in a discrepancy of over 200,000 births, corresponding to approximately 12% of the live births analyzed. Additionally, the analysis identified nearly 3,000 cases (28.8% of cases) of ACSCV. These discrepancies, which were not addressed or considered by the authors, may be explained by migratory issues inherent in the search for better health care, unscheduled transit, or health tourism, and the Search for lower-cost housing (considering the regions where the cases were identified) or better access to public health services in the region.

3. The data does not support the argument or can be easily refuted. However, it is possible to revise the analysis for those who are actually residents or extrapolate the discussion to data on non-resident births. It is imperative to incorporate accurate information in the methodology.

4. Issues of lesser relevance: It is important to note that certain English words are not italicized. The term “Queen and Shopfile” is employed to denote a specific arrangement of items.

References:

1.ALMEIDA-FILHO, Naomar De; BARRETO, Mauricio Lima. Desenhos de Pesquisa em Epidemiologia. In: _ (Org.). . Epidemiologia & Saúde: fundamentos, métodos, aplicaçoes. Rio de Janeiro: Guanabara Koogan, 2012. p. 165-174

2. Sun HY. Prenatal diagnosis of congenital heart defects: echocardiography. Transl Pediatr. 2021 Aug;10(8):2210-2224. doi: 10.21037/tp-20-164. PMID: 34584892; PMCID: PMC8429868.

3. Mortality for Critical Congenital Heart Diseases and Associated Risk Factors in Newborns. A Cohort Study. Arquivos Brasileiros de Cardiologia, v. 111, p. 666, 2018. https://doi.org/10.5935/abc.20180203

4. Risk factors for critical and complex congenital heart diseases: Case-control study. Progress in Pediatric Cardiology. https://doi.org/10.1016/j.ppedcard.2022.101612

